# Estimating county-level dental care utilization among adults in California using multilevel modeling with raking approach

**DOI:** 10.1186/s13690-025-01673-6

**Published:** 2025-07-11

**Authors:** Yilan Huang, Honghu Liu

**Affiliations:** 1 Department of Biostatistics, Fielding School of Public Health, University of California, Los Angeles, Los Angeles, CA USA; 2 Section of Public and Population Health, School of Dentistry, University of California, Los Angeles, Los Angeles, CA USA; 3 Department of Medicine, David Geffen School of Medicine, University of California, Los Angeles, Los Angeles, CA USA

**Keywords:** Small area Estimation, Raking, Multilevel model, Dental care utilization

## Abstract

**Background:**

Regular dental visits are essential for oral health, yet disparities between regions exist due to socioeconomic and geographic factors. While national surveys provide valuable data on dental care utilization, they generally lack sufficient sample sizes at the local level to generate reliable county-level estimates. Small area estimation techniques, such as multilevel regression and post-stratification (MRP), can help address this gap by producing robust estimates for smaller geographic areas. However, the MRP approach relies on detailed population data in the form of joint distributions and cannot be applied when only marginal distributions are available.

**Methods:**

This paper introduces a hybrid approach combining multilevel modeling with the raking procedure. We used individual-level data from the 2018 Behavioral Risk Factor Surveillance System (BRFSS) and census data from American Community Survey to estimate county-level dental care utilization among adults in California.

**Results:**

The county-level dental care utilization in California ranged from 52.5 to 73.1%, with a median of 63.1%. Our model-based estimates matched direct BRFSS estimates at metropolitan and micropolitan statistical area levels. Furthermore, we found significantly positive correlations between our model-based estimates and direct estimates from the California Health Interview Survey for 41 counties (Pearson coefficient: 0.801, *P* < 0.001).

**Conclusions:**

The proposed approach accounts for individual- and area-level factors while overcoming data constraints that limit the application of MRP. The findings demonstrate the feasibility of this approach in generating county-level estimates, supporting public health planning and targeted interventions to reduce disparities in dental care utilization.

**Supplementary Information:**

The online version contains supplementary material available at 10.1186/s13690-025-01673-6.

**Table:** 

**Text box 1. Contributions to the literature**
• The multilevel regression and post-stratification approach for small area estimation requires joint distributions of the weighting variables in the population and cannot be applied when only margin distributions are available.
• This study introduces a new small area estimation (SAE) method that combines multilevel regression with the raking procedure and demonstrates the practical application of this approach by generating county-level estimates of dental care utilization in California.
• Our approach enhances the applicability of multilevel regression models for SAE in public health research and offers a flexible solution for generating reliable local-level estimates in regions where fine-grained census data are not available.

## Introduction

Regular dental visits are essential for maintaining good oral health, as they enable preventive care, early detection of dental and oral diseases, and timely intervention [1]. Among adults aged 18 and older, the likelihood of having a dental visit in the past 12 months varies substantially by socio-demographic factors, such as income, education, and insurance status, and by geographic region [2, 3]. Factors such as age, race, sex, and rural versus urban residence further contribute to disparities in dental care access and utilization [4, 5]. These disparities in dental visits not only reflect inequities in access to care but are also linked to disparities in oral health outcomes, with lower utilization often associated with higher rates of dental caries, periodontal disease, and tooth loss [6]. Understanding patterns of dental service utilization is crucial for identifying target populations for oral health interventions, especially as public health initiatives aim to address barriers and promote equitable access to dental care for all adults [7–9].

Several studies have underscored substantial disparities in dental care utilization. For instance, data from the Medical Expenditure Panel Survey (MEPS) indicated that about 42% of adults aged 18–64 had a dental visit in 2019, with significantly lower utilization rates among the uninsured population [10]. Additionally, analysis of the National Health Interview Survey (NHIS) revealed that adults residing in rural areas were less likely to have had a dental visit in the past year compared to their urban counterparts in 2019 and 2020, which reflected geographic disparities in dental care use [11]. However, few studies have produced reliable estimates of dental care utilization at the local level. This gap presents a critical challenge for local health departments seeking to reduce disparities and improve oral health outcomes through targeted, data-driven interventions.

Area-specific oral health data are needed to assist state, county and local health departments in conducting community needs assessments and developing community health improvement plans. However, while national health surveys in the U.S. provide a critical cost-effective way to generate suitable statistics for measuring and monitoring national or state population health, they often do not have statistically sufficient samples to produce direct survey estimates for most counties or local areas. Small area estimation (SAE) is a statistical technique used to produce statistically reliable estimates for smaller geographic areas than those for which the original national health surveys were designed. To meet the growing need for local-level data in public health practice, various small area estimation methods, especially model-based approaches, have been applied to produce SAEs using data from national health surveys, such as the National Health and Nutrition Examination Survey (NHANES), the National Health Interview Survey (NHIS), and the National Survey of Children’s Health (NSCH) [12–15]. The Behavioral Risk Factor Surveillance System (BRFSS) has been a major data source used to produce model-based SAEs at the levels of the county, zip code, and census tract [16], due to its extensive geographic coverage, large sample size, and diverse health-related variables. These features of BRFSS allow researchers to generate precise local health estimates, supporting targeted public health interventions and resource allocation.

The principle underlying model-based SAE is to build statistical models on survey data while borrowing strength from other data sources, such as population registry or census data, to make small area inferences [17]. Multilevel modeling offers a unique model-based framework to understanding the impacts of individual- and area-level factors on health outcomes. The multilevel regression and post-stratification (MRP) approach, originally developed by Gelman and Little in 1997 [18], is a model-based method that combines hierarchical regression with population-level post-stratification to produce reliable estimates for small geographic areas or subpopulations using survey data. Recent studies have applied the MRP approach to national health survey data to generate SAE of selected health indicators, such as obesity and smoking, at state, county, and census block levels [15, 19]. However, MRP presents several estimation challenges and requirements, particularly regarding the availability of joint distribution for all weighting variables in the population. This data requirement makes it infeasible to apply MRP in regions where such data are generally not available, either due to data protection laws or because the data are not gathered by a single agency [20]. Furthermore, even when such census data are available, researchers are limited to using only demographic variables that are provided in the form of joint distributions as predictors for their multilevel regression models.

The aim of this paper is to develop a hybrid technique that combines multilevel regression models with the raking approach, to utilize the strength of the multilevel regression approach in situations where joint distributions cannot be obtained. We demonstrate a practical application of this method to generate SAEs of dental care utilization at county level for adults in California using BRFSS data and census data. To evaluate and validate the model-based estimates, we perform validation by comparing the SAEs with direct estimates from BRFSS and available county-level estimates from a state health survey.

## Methods

### Survey data: 2018 SMART BRFSS data

The BRFSS is a nationwide, state-based random-digit-dialing telephone survey of the noninstitutionalized U.S. adult population aged 18 years and older. The survey uses a disproportionate stratified sample design and is administered annually to households with landlines or cellular telephones by state health departments in collaboration with the Centers for Disease Control and Prevention (CDC). BRFSS collects data from individuals on health risk behaviors, preventive health practices, chronic conditions, and health care access, with a primary focus on chronic disease and injury. It also collects information on a range of demographic and health services measures. As part of the 2018 survey, all respondents were asked about dental care utilization with the question: “Including all types of dentists, such as orthodontists, oral surgeons, and all other dental specialists, as well as dental hygienists, how long has it been since you last visited a dentist or a dental clinic for any reason?”

The Selected Metropolitan/Micropolitan Area Risk Trends (SMART) project uses the BRFSS to analyze the data of selected metropolitan and micropolitan statistical areas (MMSAs) with 500 or more respondents. CDC analyzes BRFSS data for MMSAs to provide localized health information that can help public health practitioners identify local emerging health problems, plan and evaluate local responses, and efficiently allocate resources to specific needs. The 2018 SMART BRFSS included five MMSAs in California: Los Angeles-Long Beach-Anaheim (*n* = 3400), Oakland-Berkeley-Livermore (*n* = 830), Riverside-San Bernardino-Ontario (*n* = 1512), Sacramento-Roseville-Folsom (*n* = 882), and San Jose-Sunnyvale-Santa Clara (*n* = 533).

We used the 2018 SMART BRFSS data, with a derived binary variable from the survey response as the outcome variable for our model: whether the respondent had a dental visit in the past year (Yes/No). Demographic and socio-economic variables were also selected from the SMART BRFSS as individual-level covariates in the multilevel model, based on their potential association with dental care utilization.

### Census data: American community survey

The American Community Survey (ACS) is currently the largest nationwide, continuous sample survey being implemented by the U.S. Census Bureau to produce reliable estimates for cities, counties, states, and the entire country. The ACS has collected demographic, housing, social, and economic data since 2000 and information on health insurance coverage since 2008. In this study, we obtained available county-level marginal population distributions of the socio-demographic variables from the 2018 five-year ACS estimates for the population aged 18 years and older, which included the population joint distribution by age and sex, population margins by educational attainment, and by race/ethnicity. The county-level percentage of population in poverty was also obtained from the ACS and used as an area-level factor for the multilevel model.

### Statistical approach: multilevel model

Let $\:y$ be a binary outcome, with $\:y=1\:$for an adult who had a dental visit in the past year and $\:y=0$ otherwise, and $\:p$ be defined as the probability of $\:y=1$; the multilevel regression model has the following form:

$$\:\text{logit}\left({p}_{ij}\right)={\varvec{x}}_{ij}\varvec{\beta\:}+{\varvec{z}}_{i}\varvec{\eta\:}+{\mu\:}_{i}$$

where $\:{p}_{ij}$ is the probability for an adult who had a dental visit in the past year in demographic group $\:j$ in county $\:i$, $\:{\varvec{x}}_{ij}$ is the vector of individual-level covariates (e.g., age, gender, education level, health insurance status, and race/ethnicity), and $\:{\varvec{z}}_{i}$ is the vector of area-level covariates (e.g., county-level percentage of population in poverty, and county-level dentist population ratio). $\:\varvec{\beta\:}$ and $\:\varvec{\eta\:}$ are the corresponding regression coefficients. $\:{\mu\:}_{i}$ represents the county-level random effect, which is assumed to be normally distributed.

### Multilevel regression with raking approach

Using the multilevel regression with raking approach, we estimated the county-level dental care utilization among adults for all 58 counties in California. Our methodological approach with the SMART BRFSS data involved the following five basic steps: (1) construct a multilevel model using SMART BRFSS data; (2) apply the multilevel prediction model to each socio-demographic group; (3) estimate the population size for each socio-demographic group based on the marginal population distributions using the raking algorithm; (4) generate model-based SAEs via post-stratification; and (5) validate model-based SAEs.

Table 1 provides an example that further illustrates the data requirements and steps involved in our method. Assume that there are eight socio-demographic groups in county $\:i$ (four education categories and two sex categories: 4 × 2 = 8). The notation $\:i\left(jk\right)$ refers to the group defined by education $\:j$ ($\:j=1-4$) and sex $\:k$ ($\:k=1,\:2$) within county $\:i$. Let $\:{p}_{i\left(jk\right)}\:$represent the probability of the outcome for an individual in group $\:i\left(jk\right)$. For post-stratification, the MRP approach requires joint distribution in each socio-demographic group, that is, the frequency of each cell ($\:{N}_{i\left(11\right)}$, $\:{N}_{i\left(12\right)}$,…, $\:{N}_{i\left(24\right)}$). However, when only the marginal distributions for sex and education level ($\:{N}_{i(j+)}$ and $\:{N}_{i(+k)}$) are available, but the joint distributions $\:{N}_{i\left(jk\right)}$ are unknown, our proposed method can address this limitation as follows:

1. A multilevel logistic regression model is used to regress the outcome on the socio-demographic variables, as described in the previous subsection.
2. The conditional probabilities $\:{\widehat{p}}_{i\left(jk\right)}$ are estimated using the estimated parameters from the multilevel logistic regression model for each group.
3. Estimate the weighting cell population sizes $\:{\widehat{N}}_{i\left(jk\right)}$ for each cell based on the marginal population distribution, using the raking procedure.
4. Finally, the overall prevalence $\:{p}_{i}$ is estimated by

$${\hat p_i} = \frac{1}{{{N_i}}} \cdot \:\sum\limits_{j = 1}^J {\sum\limits_{k = 1}^K {{{\hat N}_{i\left( {jk} \right)}}\: \cdot \:{{\hat p}_{i\left( {jk} \right)}}.} }$$

**Table 1:** An illustrative example of the data requirements for multilevel regression approaches

		Education level				
		No high school	High school	College	Postgraduate	Total
Sex	Male	$\:{N}_{i\left(11\right)}$	$\:{N}_{i\left(12\right)}$	$\:{N}_{i\left(13\right)}$	$\:{N}_{i\left(14\right)}$	$\:{N}_{i(1+)}$
	Female	$\:{N}_{i\left(21\right)}$	$\:{N}_{i\left(22\right)}$	$\:{N}_{i\left(23\right)}$	$\:{N}_{i\left(24\right)}$	$\:{N}_{i(2+)}$
	Total	$\:{N}_{i(+1)}$	$\:{N}_{i(+2)}$	$\:{N}_{i(+3)}$	$\:{N}_{i(+4)}$	$\:{N}_{i}$

In this study of dental care utilization, we stratified the population into 240 socio-demographic groups based on age-sex (6 × 2 categories), education level (4 categories), and race/ethnicity (5 categories). The multilevel model used for the main analysis in this study (Model 1) included age, sex, education level, and race/ethnicity at individual level, percent of population below poverty level at county level, and the county-level and residual random effects. Multilevel regression models were fitted in R 4.4.2, using the lme4 package [21]. We used the estimated model parameters to predict the individual-level probability of dental care utilization for each group and applied a bootstrap approach with 1,000 resamples to generate 95% confidence intervals (CIs). Population counts for each group within each county were estimated using the raking algorithm with marginal population distributions from the 2018 census data, using the ipfr package in R. Finally, county-level dental care utilization rates were generated by weighting the individual-level probability using the estimated population counts.

### Internal and external validation

We implemented internal validation of our model-based SAEs by comparing them with 2018 SMART BRFSS direct estimates for all five MMSAs in California. For external validation, we compared our county-level model-based estimates with the 2017–2018 California Health Interview Survey (CHIS) direct survey estimates, which were available for 41 California counties. Basic summary statistics (minimum, first quartile, median, third quartile, maximum, interquartile range, and range) were used to compare the distributions of our model-based SAEs and CHIS direct estimates. We also calculated the Pearson correlation and Spearman Rank correlation coefficients of the estimates to evaluate their internal consistency.

### Model comparisons

Model 2 and Model 3 were fitted with different individual- and area-level covariates compared to Model 1:

- *Model 1 (Main Model)*: included all individual-level covariates (age, sex, education level, and race/ethnicity), the percent of population below poverty level at the county level, as well as the county and residual random effects.
- *Model 2*: included individual-level covariates (age, sex, and education level), the percent of population below poverty level at the county level, and the county and residual random effects.
- *Model 3*: included individual-level covariates (age, sex, and education level), and the county and residual random effects.

The raking procedure was then applied to estimate the population size of each socio-demographic group, using the individual-level covariates in each model as margins. We compared the county-level estimates generated by the three models to assess the performance of the multilevel regression with raking approach under varying data availability of auxiliary variables at individual and area levels. In addition, we used mean squared error and mean absolute difference to evaluate the consistency between the model-based estimates ($\:{m}_{i}$) and direct survey estimates ($\:{d}_{i}$). The mean squared error was calculated as $\:1/N\sum\:_{i=1}^{N}{({m}_{i}-{d}_{i})}^{2}$, and the mean absolute difference as $\:1/N\sum\:_{i=1}^{N}\left|{m}_{i}-{d}_{i}\right|$, where $\:N$ is the number of small areas in the comparison.

## Results

### Model-based county-level estimates

**Fig. 1:**
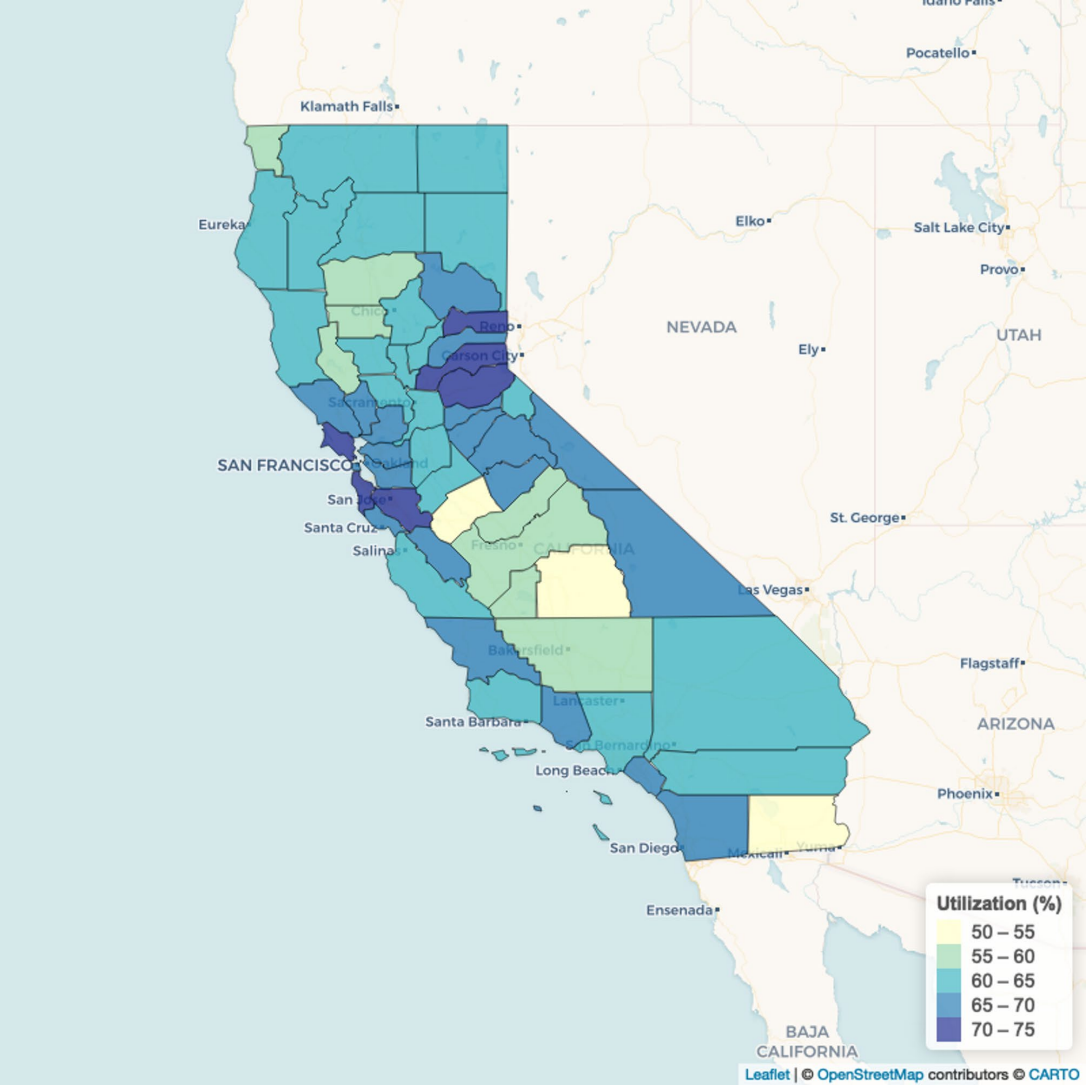
Model-based county-level estimates of the percentage of adults who had a dental visit in the past year, 2018

Figure 1 shows model-based county-level estimates of the percentage of adults aged 18 years and older who visited a dentist or dental clinic in the past year across California, generated using Model 1 (Main Model). The map illustrates variation in the estimated rate across counties, ranging from a minimum of 52.5% in Tulare County to a maximum of 73.1% in Marin County, with a median of 63.1% and an interquartile range of 7.1%.

### Internal validation

Table 2 compares model-based estimates and direct estimates from the 2018 SMART BRFSS for the percentage of adults aged 18 years and older who visited a dentist or dental clinic in the past year across five MMSAs in California. The model-based percentage estimates closely aligned with direct estimates, and all were contained in the 95% CI of the corresponding direct estimates. For instance, in Los Angeles-Long Beach-Anaheim, the model-based estimate was 65.0% (95% CI: 62.3–67.5%), while the direct estimate was 66.6% (95% CI: 64.4–68.8%). Similarly, in San Jose-Sunnyvale-Santa Clara, the model-based estimate of 71.0% (95% CI: 67.7–74.3%) was slightly lower than the direct estimate of 72.9% (95% CI: 67.9–77.9%). The model-based and direct estimates showed similar patterns of geographic variation, with consistent rankings across the five MMSAs.

**Table 2:** Comparisons of direct estimates from SMART BRFSS and model-based estimates of the percentage of adults aged 18 years and older who had a dental visit in the past year, 2018

MMSA	Model-based estimate			2018 SMART BRFSS direct estimate		
	**Utilization** (%)	**95% CI**		**Utilization** (%)	**95% CI**	
Los Angeles-Long Beach-Anaheim	65.0	62.3	67.5	66.6	64.4	68.8
Oakland-Berkeley-Livermore	69.6	66.8	72.4	71.4	67.3	75.5
Riverside-San Bernardino-Ontario	62.8	59.7	65.7	64.8	61.7	68.0
Sacramento-Roseville-Folsom	68.3	65.7	70.8	70.9	67.1	74.8
San Jose-Sunnyvale-Santa Clara	71.0	67.7	74.3	72.9	67.9	77.9

### External validation with direct County estimates from CHIS

**Fig. 2:**
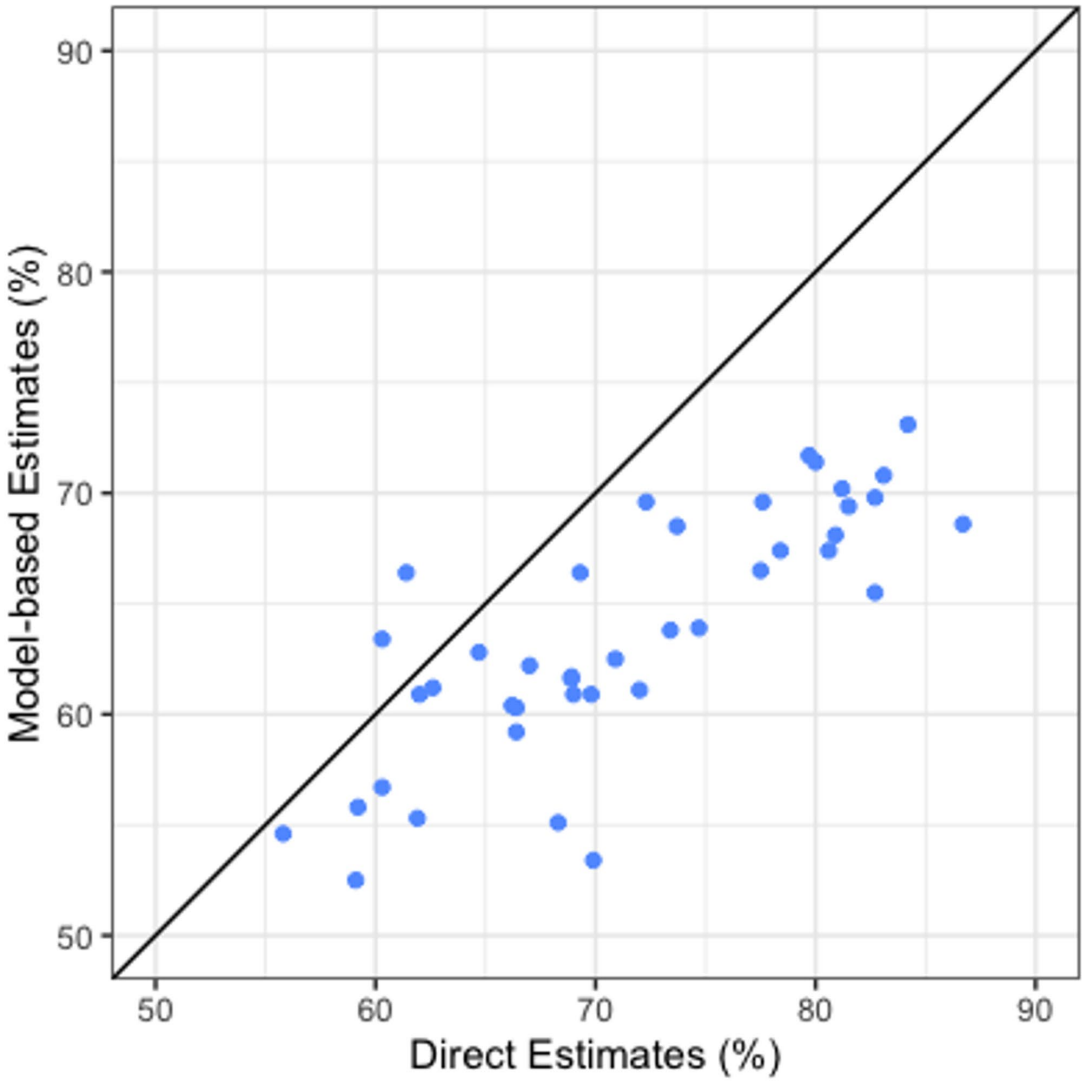
Comparison of model-based county-level estimates and CHIS direct county-level estimates of the percentage of adults aged 18 years and older who had a dental visit in the past year, 2018.* * Included 41 counties for which 2018 CHIS direct estimates were available

For 41 counties with available county-level estimates in the 2018 CHIS, moderate positive correlations were found between model-based estimates of rate of dental clinic visit in the past year and the CHIS direct estimates (Pearson correlation = 0.801, *P* < 0.001; Spearman rank correlation = 0.805, *P* < 0.001) (Fig. 2; Table 3). 90% of counties (18 out of 20) with direct estimates higher than the median also ranked above the median when using model-based estimates. The direct estimates were generally higher than the corresponding model-based estimates. Figure S1 (a) and (b) illustrate quartile distribution of the model-based estimates and CHIS direct estimates, which show similar geographic clustering of the higher and lower levels of dental care utilization.

**Table 3:** Correlation coefficients and summary statistics for model-based county-level estimates and CHIS direct county-level estimates of the rate of dental clinic visit in the past year among adults aged 18 years and older, 2018.*

	Pearson correlation	Spearman rank correlation	Utilization (%)					IQR (%)
			Min	25%	Median	75%	Max	
Model-based estimates	0.801 (*P* < 0.001)	0.805 (*P* < 0.001)	52.5	60.9	63.4	68.5	73.1	7.6
CHIS direct estimates			55.8	66.2	69.9	79.7	86.7	13.5

* Included 41 counties for which 2018 CHIS direct estimates were available

### Model comparisons

**Fig. 3:**
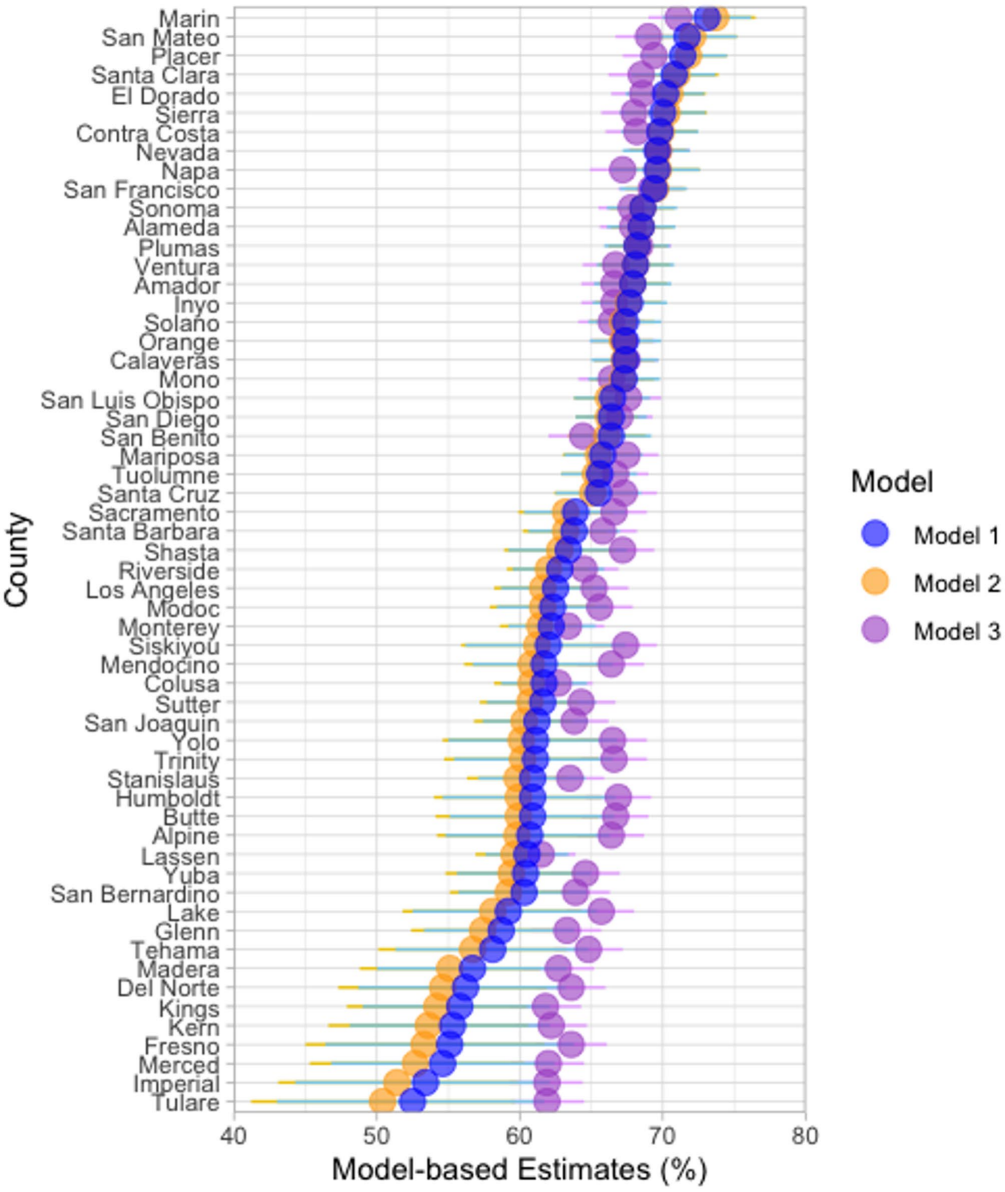
Comparison of model-based county-level estimates of the percentage of adults aged 18 years and older who had a dental visit in the past year, 2018. Horizontal lines represent the 95% confidence intervals

Figure 3 compares county-level dental utilization estimates generated by three SAE models, ordered from highest to lowest rate according to the Model 1 estimates. Across the counties, Model 1 (blue) and Model 2 (orange) generated similar estimates, and Model 3 (purple) generally produced estimates with narrower confidence intervals compared to Model 1 and Model 2. Model 3 also showed less variation across counties, with a smaller range of estimates: 52.5–73.1% for Model 1, 50.4–73.7% for Model 2, and 61.5–71.1% for Model 3.

According to the internal validation (Table S1), for the five MMSAs in California, all model-based estimates fell within the 95% CIs of the corresponding BRFSS direct estimates. On the other hand, the 95% CIs of the Model 3 estimates did not contain the BRFSS direct estimates for three of the MMSAs, compared to only one MMSA for Models 1 and 2. For the MMSA where none of the model-based 95% CIs included the BRFSS direct estimate (i.e., Sacramento-Roseville-Folsom), Model 1 produced the estimate closest to the BRFSS value. When benchmarked against the BRFSS direct estimates, Model 1 had the lowest mean squared error (4.0%) and mean absolute difference (2.0%), followed by Model 2 (4.8% and 2.1%, respectively) and Model 3 (8.2% and 2.6%, respectively).

## Discussion

In this paper, we combined multilevel modeling with the raking approach and generated county-level estimates of the rate of dental visits in the past year among adults in California in 2018. The county-level estimates revealed geographic disparities in dental care utilization across California. Urban counties such as Alameda (68.5%), Contra Costa (69.8%), Marin (73.1%), San Francisco (69.4%), San Mateo (71.7%), and Santa Clara (70.8%) exhibited relatively higher rates of dental visits. In contrast, many rural counties, including Imperial (53.4%), Merced (54.6%), and Madera (56.7%), showed lower dental visit rates. These findings align with the existing literature indicating that rural residents faced greater barriers to dental care, resulting in lower utilization [22]. Such barriers often include limited availability of dental professionals, lack of adequate transportation, poor oral health education, and higher rates of uninsured populations in rural areas [22, 23]. Overall, the model-based estimates showed high internal validation performance at the MMSA level and high correlations with county-level direct estimates from CHIS. Our small area estimates can help the state and local oral health programs focus on areas of low dental visits rate that may benefit from interventions to increase dental care utilization.

We observed that in the internal validation the model-based estimates were consistently lower than the BRFSS direct estimates, which may indicate potential bias in the model. To understand and address this issue, further research could incorporate additional individual-level predictors, such as dental health insurance coverage, which is an important determinant of dental care utilization. Including relevant area-level factors, such as the county-level dentist-to-population ratio available from the Area Health Resources Files, may further enhance model accuracy by capturing variation in local healthcare resource availability. Additionally, since the current study employed unweighted analyses, further work could explore the differences between unweighted and weighted model-based estimates derived from BRFSS data. Specifically, comparing multilevel regression models fitted with and without BRFSS survey weights would provide valuable insights into the impact of survey weighting on the precision and reliability of small area estimates with the proposed approach.

With the external validation, for most of the counties with available direct estimates, CHIS estimates were higher than our model-based estimates. The discrepancy can be attributed to the differences in survey design, sampling, and measurements. The 2017–2018 CHIS included a statewide landline random-digit-dialing (RDD) sample and a statewide cell phone sample, and each sample made up 50% of obtained interviews [24]. To better capture California’s diverse population, CHIS oversampled some race/ethnic groups, such as Vietnamese and Koreans, and interviews were conducted in six languages (English, Spanish, Chinese, Vietnamese, Korean, and Tagalog), which potentially lead to higher utilization estimates. On the other hand, BRFSS used a RDD telephone survey that included both landlines and cell phones, but employed a disproportionate stratified sample design, and conducted interviews only in English and Spanish. Both BRFSS and CHIS used computer-assisted telephone interviewing system, but differences in interviewer training, question order, or survey administration procedures could still result in systematic differences in responses. Additionally, CHIS employed a multi-step weighting methodology with population control totals primarily from the California Department of Finance’s population estimates [25], while our model-based estimates relied on the standard raking procedure and population census data from the American Community Survey. These factors indicate the challenges in comparing estimates from different data sources and emphasize the need for careful interpretation of small area estimates in the context of external validation.

Comparison of the three SAE models revealed that Models 1 and 2 produced more similar estimates, which suggests that the county-level poverty rate plays a substantial role in explaining the variation in dental utilization estimates. In contrast, Model 3, which did not include county-level poverty rate, produced estimates that had smaller geographic variation and lower consistency with the BRFSS direct survey estimates. Table S1 also presents MMSA-level estimates generated by a fixed-effect model (Model 4), which included individual-level covariates and treated MMSA as a fixed effect. While Model 4 produced estimates that were generally close to the survey direct estimates, Model 1 showed slightly better overall performance, as indicated by lower mean squared error and mean absolute difference. The observed variability in the estimates across the models indicates the importance of selecting an appropriate SAE model to ensure robust and accurate estimation. Generally, model specification should be informed by empirical evidence of relationships between covariates and the outcome of interest, as well as the availability and quality of auxiliary data at the individual and area levels. In practice, model design should also consider the trade-off between model complexity and interpretability to produce reliable estimates that are accessible to policymakers and practitioners for informing policy development and resource allocation.

Raking is a calibration procedure used to adjust the counts in a contingency table to match the known marginal distributions. It is commonly employed in survey weights adjustments to reduce nonresponse and noncoverage biases. In this study, we applied the raking approach to estimate the population count for each demographic-geographic respondent subgroup involved in the multilevel modeling, using known marginal population distributions. The resulting joint distribution was then used as weights, which, when combined with the parameter estimates from the multilevel modeling, allowed us to produce an overall population-level estimate for the small area of interest. This innovative hybrid technique retains the advantage of MRP, which allows the use of information from both individual-level data within the survey sample and from various area-level covariates external to the original sample. Furthermore, it addresses the key requirement of MRP for the population size data in all cross-classification cells.

In recent years, several methods have been proposed for multilevel regression approaches that use margins only. For example, Kastellec et al. (2015) use data from multiple surveys to construct synthetic joint distributions [26]. However, this approach is limited to the rare practical cases where multiple surveys are available. Leemann and Wasserfallen (2017) propose a method that applies the multilevel regression approach with population margins in the case when only one survey is available [20]. In their method, the joint distribution of the weighting variables is estimated by a simple multiplication of the marginal distributions, which assumes that the weighting variables are independent, a condition that is rarely satisfied in practical applications. In contrast, our hybrid approach overcomes these limitations by applying the raking algorithm and generates joint distributions without requiring multiple surveys or the assumption of independence. This flexibility makes it applicable to a broader range of practical scenarios, including cases with complex dependencies between variables. Bruch and Felderer (2023) propose two approaches that combine the multilevel regression method with weighting algorithms when only marginal population distributions are available [27]. Their first approach combines multilevel regression with the raking procedure, however, applies only to cells with elements in the sample. Their second approach introduces a combination of the multilevel logistic regression and the population cell size estimation method, but primarily uses the weighted least squares method to obtain estimates of the population joint distributions. In our study, we explore the possibility for extending the raking procedure to all weighting cells, with or without sampling elements, and apply this approach using real-world data.

Our study includes a few limitations. First, our analysis depended on the 2018 SMART BRFSS data, which only included samples from five MMSAs in California. It is possible that the relationship between dental visits and individual-level demographics differs in some other counties. Consequently, the generalizability of our results to other regions within California may be limited. Second, in our multilevel model, in addition to the county population composition, we used county-level random effects to capture the impact of factors such as social, economic, cultural and policy differences on the variation in dental care utilization between counties. To better understand the variation, further in-depth exploration of socioeconomic factors, access to care, and local health programs is needed. Last, our approach shares the same characteristic with standard raking procedure regarding the preference for independent margins. It seems that further research should be conducted on how to implement the approach in practical applications and how to improve the approach with more complex models that include potential interactions.

## Electronic supplementary material

Below is the link to the electronic supplementary material.

Supplementary Material 1

Supplementary Material 2

## Data Availability

The SMART BRFSS data can be accessed at https://www.cdc.gov/brfss/smart/Smart_data.htm. Census data is available at https://data.census.gov/. The direct estimates from the California Health Interview Survey (CHIS) were obtained through the online query system, AskCHIS™, which can be accessed at https://healthpolicy.ucla.edu/our-work/california-health-interview-survey-chis/access-chis-data.

## Abbreviations

MEPS: Medical Expenditure Panel Survey
NHIS: National Health Interview Survey
SAE: Small area estimation
NHANES: National Health and Nutrition Examination Survey
NSCH: National Survey of Children’s Health
BRFSS: Behavioral Risk Factor Surveillance System
MRP: Multilevel regression and post-stratification
CDC: Centers for Disease Control and Prevention
SMART: Selected Metropolitan/Micropolitan Area Risk Trends
MMSA: Selected metropolitan and micropolitan statistical area
ACS: American Community Survey
CI: Confidence interval
CHIS: California Health Interview Survey
RDD: Random-digit-dialing
