# Supplementary material for: Estimating county-level dental care utilization among adults in California using multilevel modeling with raking approach
Source: Arch Public Health. 2025 Jul 11;83:183. doi: 10.1186/s13690-025-01673-6 (PMC12247392; doi:10.1186/s13690-025-01673-6)
Supplement: Supplementary file 2 — Supplementary Material 2 [file 13690_2025_1673_MOESM2_ESM.docx]

**Table S1.** Model comparisons: model-based estimates of dental care utilization (%) and 95% CIs at the MMSA level.

| MMSA | Model 1 | Model 2 | Model 3 | Model 4* | 2018 SMART BRFSS direct estimate |
| --- | --- | --- | --- | --- | --- |
| Los Angeles-Long Beach-Anaheim | 65.0  (62.3-67.5) | 65.0  (62.8-67.2) | 65.4  (62.9-68.2) | 65.3  (62.4-68.1) | 66.6  (64.4-68.8) |
| Oakland-Berkeley-Livermore | 69.6  (66.8-72.4) | 69.7  (67.1-72.4) | 68.3  (65.3-70.5) | 69.3  (65.5-73.1) | 71.4  (67.3-75.5) |
| Riverside-San Bernardino-Ontario | 62.8  (59.7-65.7) | 62.4  (59.7-65.0) | 63.3  (61.5-67.0) | 62.1  (58.6-65.6) | 64.8  (61.7-68.0) |
| Sacramento-Roseville-Folsom | 68.3  (65.7-70.8) | 67.6  (65.4-69.7) | 67.6  (64.5-69.7) | 69.0  (65.1-72.6) | 70.9  (67.1-74.8) |
| San Jose-Sunnyvale-Santa Clara | 71.0  (67.7-74.3) | 71.5  (68.2-74.6) | 68.8  (66.0-71.0) | 70.5  (65.8-74.9) | 72.9  (67.9-77.9) |
| Mean squared error** | 4.0 | 4.8 | 8.2 | 4.6 | N/A |
| Mean absolute difference** | 2.0 | 2.1 | 2.6 | 2.1 | N/A |

* Model 4: included individual-level covariates (age, sex, education level, race/ethnicity) and treated MMSA as a fixed effect.

** Used BRFSS survey direct estimates as benchmarks.


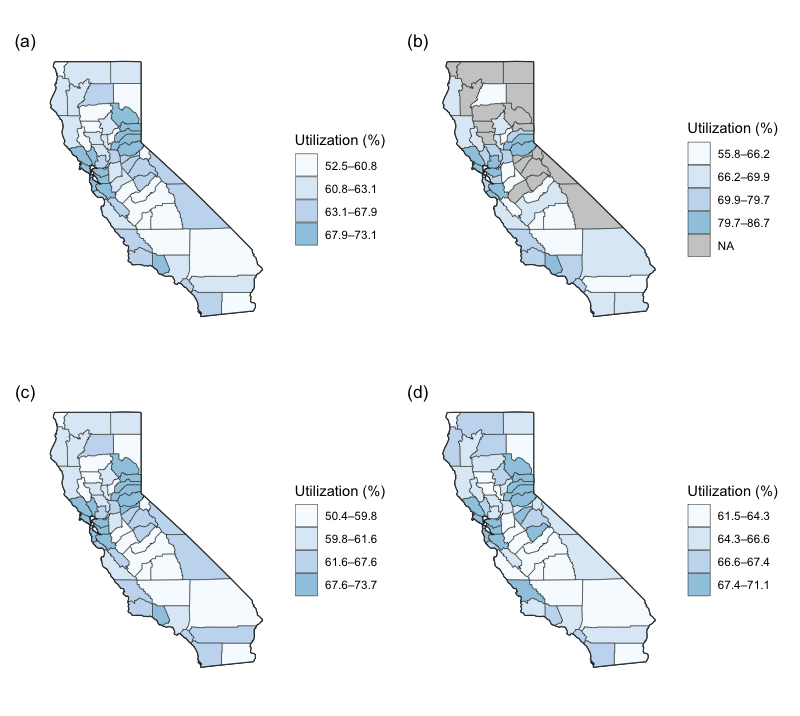


**Figure S1.** County-level estimates of the rate of dental clinic visit in the past year among adults aged 18 years and older in California, 2018: (a) Model 1, (b) CHIS direct estimates, (c) Model 2, (d) Model 3. Colors represent the quartile distribution of estimates generated by each corresponding model or method.
